# Putting the ‘Art’ Into the ‘Art of Medicine’: The Under-Explored Role of Artifacts in Placebo Studies

**DOI:** 10.3389/fpsyg.2020.01354

**Published:** 2020-07-22

**Authors:** Michael H. Bernstein, Cosima Locher, Tobias Kube, Sarah Buergler, Sif Stewart-Ferrer, Charlotte Blease

**Affiliations:** ^1^Center for Alcohol and Addiction Studies, School of Public Health, Brown University, Providence, RI, United States; ^2^School of Psychology, University of Plymouth, Plymouth, United Kingdom; ^3^Division of Clinical Psychology and Psychotherapy, Faculty of Psychology, University of Basel, Basel, Switzerland; ^4^Division of General Medicine, Beth Israel Deaconess Medical Center, Harvard Medical School, Boston, MA, United States; ^5^Pain and Psychotherapy Research Lab, University of Koblenz-Landau, Landau, Germany; ^6^Research Unit of General Practice, University of Southern Denmark, Odense, Denmark; ^7^School of Psychology, University College Dublin, Dublin, Ireland

**Keywords:** placebo, placebo effects, expectancy, mindset, psychology, classical conditioning

## Abstract

Research in social psychology demonstrates that physical environmental factors – or “artifacts” such as provider clothing and office décor – can influence health outcomes. However, the role of artifacts in augmenting or diminishing health outcomes is under-explored in the burgeoning discipline of placebo studies. In this paper, we argue that a careful consideration of artifacts may carry significant potential in informing how placebo effects can be maximized, and nocebo effects minimized in clinical settings. We discuss the potential mechanisms, including classical conditioning, response expectancy, and mindsets, by which artifacts might enhance or diminish these effects. Next, we propose testable hypotheses to investigate how placebo and nocebo effects might be elicited by artifacts in care settings, and conclude by providing innovative research designs to advance this novel research agendum.

## Introduction

From the soft-furnishings in the waiting area, to the artwork adorning the walls, to the physician’s framed credentials: as soon as a patient enters the clinical environment he or she is unavoidably seeped in a range of artifacts. To date, a range of studies in social and health psychology demonstrates that these physical environmental factors – or “artifacts” – can influence patients’ perceptions about the quality of their care, and, as a result, even their health outcomes (e.g., Arneill and Devlin, 2002; Devlin et al., 2009; Petrilli et al., 2018). In this paper, we propose that artifacts, which have been investigated at some length in psychology, deserve further scrutiny within the burgeoning field of placebo studies. Placebo effects have been recognized for centuries (Findley, 1953; Shapiro, 1959, 1960). Thomas Jefferson referred to placebos as a “pious fraud” (De Craen et al., 1999); according to Shapiro (1968, p. 667), Stanley Hall, the founding president of the American Psychological Association, once exclaimed, “Physicians appeal to the imagination in desperate cases with bread pills and placeboes [*sic*].” Placebo effects refer to psychobiological processes that give rise to genuine therapeutic effects for a range of conditions (Blease, 2019). In the last three decades research on placebo studies has increased considerably. Scientific findings now show that placebo effects are powerful (Finniss et al., 2010), particularly for “self-appraised symptoms” (Kaptchuk and Miller, 2015). It is now understood that placebo effects are particularly relevant for many commonly presented symptoms and conditions including depression, anxiety, and pain (Kaptchuk and Miller, 2015). Nocebo effects, on the other hand, refer to psychobiological processes that engender adverse health outcomes (Colloca and Barsky, 2020).

Prior research in placebo studies largely focused on (a) *what* providers say, (b) *how* they say it, or (c) the nature of treatment in influencing placebo and nocebo effects. With regard to the content of provider disclosures, research suggests that positively valenced information, and a convincing rationale may augment placebo effects (Thomas, 1987; Locher et al., 2017). With regard to the how information is disclosed, considerable research in placebo studies has examined social and environmental factors in the context of the interaction between a patient and caretaker (Kaptchuk et al., 2008; Leibowitz et al., 2019). Finally, studies also show that the contingent features of placebo treatments such as taste, color, size, or invasiveness can also modulate the effect (Branthwaite and Cooper, 1981; De Craen et al., 1996; Kaptchuk et al., 2008; Kam-Hansen et al., 2014; Faasse et al., 2016; Berna et al., 2017).

However, very little attention has been given to *non-medical artifacts* arising in the proximal clinical environment. Artifacts have been referenced in the history of psychology. Vygotsky (1962, 1978) first discussed the notion of artifacts as culturally charged tools and symbols, theorizing their influence on learning and human behavior. Artifacts are also described in the theoretical framework of Cultural-Historical Activity Theory (Engeström, 1999; Foot, 2014). Our discussion of artifacts is rooted in present-day cognitive science. From this perspective, we also draw on theories about the role of contextual factors in psychotherapy. With regard to the latter Frank and Frank (1991) articulate the importance of the “healing setting” in psychotherapy outcomes which, they argue, includes “symbol[s of] the therapist’s role as a healer” (p. 41). According to Frank and Frank, these symbols can include “bookshelves, (an) impressive desk, couch, and easy chair” (p. 41). By taking a cognitive science perspective – rather, than a semiotics approach – we explore how these contextual factors might influence health outcomes, a consideration that so far has gone largely unexplored in experimental placebo research.

Drawing on well-established psychological findings, we discuss non-medical artifacts that we suggest placebo researchers consider as factors that potentially influence healthcare outcomes. Such artifacts include clinician clothing and office décor. Our goal in this Conceptual Analysis is therefore to propose a novel research direction in placebo studies: namely, to investigate whether non-medical artifacts in the context of care can influence the size of placebo effects.

In what follows we offer an introduction to current findings on the role of non-medical artifacts in healthcare. We focus specifically on clothing, décor, and how artifacts may influence behavior. Next, we provide an overview of research into placebo and nocebo effects. We describe the mechanisms by which placebo and nocebo effects arise, focusing on how artifacts might engender these effects among patients via non-conscious psychological processes. Linking these two distinct bodies of literature, we propose specific hypotheses, and suggest experimental designs aimed at investigating the potential of artifacts to elicit placebo effects. Finally, if artifacts play a role in modulating the size of placebo effects, we note that this will have important ethical implications for clinical practice. In short, putting the “art” into “the art of medicine” may require the medical community to expand beyond current conceptual standards of care to establish appropriate therapeutic aesthetics for health practitioners’ clothing, as well as for décor, natural lighting, and other features of the healthcare environment.

## The Influence of Artifacts on Healthcare Outcomes

### Clothing

One particular artifact that has received extensive attention in health research is physician attire. For example, Petrilli et al. (2018) surveyed over 4,000 patients in primary care, emergency department, hospital, and surgery settings at 10 United States academic hospitals. Just over half responded that the clothing their physician wears was important to them. Participants were also presented with a series of pictures of male and female doctors with manipulated attire. Overall, participants rated physicians more favorably when they were dressed formally with a lab coat. Similarly, Rehman et al. (2005) found that among 400 patients from an internal medicine outpatient clinic at a US Veterans Administration Center, 76% of respondents expressed a preference for a physician with a white coat. Further, the authors found that this preference for formal attire was associated with patients’ perceptions about physician confidence, trust, as well as their openness in discussing intimate personal problems with their doctor. One systematic review (Petrilli et al., 2015) concluded that, overall, 70% of the 30 studies included in the analysis found clear attire preferences, particularly for formal dress and white coats. The authors also reported that attire preferences were influenced by patients’ age: formal attire preference was slightly higher among older compared to younger patients. Also, in outpatient clinics attire preferences were generally higher than in acute care settings.

Moreover, cultural factors seem to influence attire preferences. Research suggests that formal attire or white coat has been preferred even more consistently in several European countries (including the United Kingdom, Belgium, and Netherlands) than in the United States (Gallagher et al., 2008; Gherardi et al., 2009; Kocks et al., 2010; Hartmans et al., 2013). Studies from Australia (Gooden et al., 2001) and Brazil (Yonekura et al., 2013) revealed a clear preference of patients for white coat, whereas patients from Saudi Arabia preferred formal attire (Al-Ghobain et al., 2012). Interestingly, in Korea, research has shown that although patients expressed a preference for white coat, traditional attire was associated with increased patient comfort with their physician (Chang et al., 2011; Chung et al., 2012).

Although experimental findings have rarely explored objective outcomes among physicians Adam and Galinsky (2012) found that physicians’ attention-span increased when donning white coats. On the other hand, Haque and Waytz (2012) have suggested that the use of white coats might, in part, have a negative impact on healthcare if, “caregivers in hospitals become anonymized a sea of white coats, which subtly diffuses their individual responsibility toward patients” (p. 177). If healthcare provider’s objective behavior changes according to what they wear, it would confound some of the results discussed here with patient perception as the outcome.

### Décor

Beyond the question of clothing, there is evidence that healthcare is influenced by aesthetic factors, consistent with the model presented by Wager and Atlas (2015). For instance, Devlin et al. (2009) have conducted a series of studies in the Northeast of the United States suggesting that the physical space where patients spend time can impact perceptions of care. In one study, college students viewed a series of pictures of physician waiting rooms (Arneill and Devlin, 2002). The authors concluded that waiting rooms that were, “nicely furnished, well-lit, contained artwork, and are warm in appearance” (p. 348) were associated with higher perceived quality of care. Similarly, Andrade et al. (2013) reported that objective quality of healthcare environments mediated patients’ perception of care. Another study, this time involving displayed credentials (i.e., diplomas) in therapists’ offices, found that the number of framed certificates on display was positively related to perceived quality of care and perceived friendliness of the therapist; interestingly, family photos did not appear to impact friendliness ratings (Devlin et al., 2009).

### Impact of Artifacts on Behavior

Beyond affecting patients’ perception of care, artifacts can influence our behavior via non-conscious processes. For example, following exposure to business-related objects participants negotiated in a more self-interested and competitive manner (Kay et al., 2004). Other research demonstrates that people keep their direct environment cleaner when sitting in a room filled with the scent of a cleaning agent (Holland et al., 2005). Even the natural environment can be considered a relevant artifact. In a naturalistic experiment, surgery patients took fewer analgesics when their hospital room window had a view of trees versus a view of a wall (Ulrich, 1984). Therefore, there is considerable potential to transfer this knowledge to a health-related environment to foster possible benefits for patients (Sheeran et al., 2013).

## Placebos and Placebo Effects

A growing body of research shows that placebo effects are therapeutically significant in the treatment of: acute (Benedetti, 2014) and chronic (Vase et al., 2014) pain, migraine (Kam-Hansen et al., 2014), major depressive disorder (Kirsch, 2014, 2019; Deacon and Spielsman, 2017), anxiety disorders (Sugarman et al., 2014), irritable bowel syndrome (Kaptchuk et al., 2008), alcohol dependence (Weiss et al., 2008), Parkinson’s Disease (Lidstone, 2014), intellectual disabilities (Jensen et al., 2017), and binge eating disorder (Blom et al., 2018).

Importantly, the term placebo should be understood in two distinctive senses that are not always distinguished by researchers (Blease, 2018; Blease and Annoni, 2019). First, in randomized controlled trials (“RCTs”), placebos are deployed as controls to test for the efficacy of particular interventions. Second, in contrast to the ethical principle of transparency, placebos are widely used by physicians for the purpose of alleviating symptoms and/or placating patients in clinical contexts (Linde et al., 2018; Bernstein et al., 2020). Placebos in this latter form occasionally constitute cellulose/sugar pills or saline injections, but more often consist of treatments, such as antibiotics or vitamins, that are not expected to have a direct curative effect on the condition being treated (Linde et al., 2018). These placebos are often referred to as “impure.” Considerable evidence demonstrates that physicians and other healthcare professional play a large role in influencing the size of placebo effects, and researchers in the field of placebo studies have begun to investigate how placebo effects might be ethically harnessed or personalized to improve patient outcomes for a variety of prevalent conditions and symptoms, including pain, depression, and irritable bowel syndrome (Enck et al., 2013; Blease et al., 2016; Evers et al., 2018).

## Nocebo Effects

Nocebo effects – often conceived as “negative placebo effects” – are usually understood as the amalgam of adverse responses to receiving an inert treatment (Kennedy, 1961). When placebos are used as controls in RCTs, patients sometimes experience side effects in the inert placebo control arm. For example, de la Cruz et al. (2010) examined the frequency of nocebo effects in patients with cancer-related fatigue. They found that 71% of patients who received an inert treatment reported nocebo effects. In RCTs, participants usually receive a list of all the potential side effects of the active drug. This might create negative expectations of treatment outcome (i.e., a nocebo effect). Similarly, patients experiencing specific negative symptoms at baseline are more likely to report side effects of the medication perhaps by misattributing them to the medication as opposed to other factors (de la Cruz et al., 2010).

Like placebo effects, nocebo effects can operate in the absence of a traditional placebo pill and comprise part of everyday treatments (Colloca and Finniss, 2012). In 2012, a systematic review concluded that nocebo responses typically result from unintended negative suggestions by physicians or the nursing staff. Such phrases may, for example, result in an unwanted focus of attention (e.g., “Are you feeling nauseous?”), in trivializing a patient’s legitimate complaint or concern (e.g., “You don’t need to worry”), or in uncertainty (e.g., “Try to take your meds regularly”), (phrases cited from Häuser et al., 2012, p. 461). Thus, the challenge in the clinic is to find a balance between the communication of important clinical information while minimizing negative instructions and a negative therapeutic context.

## Proposed Mechanisms of Placebo and Nocebo Effects

Placebo effects are understood to be genuine psychobiological events that engage in cognitive processes to promote healing. The mechanisms behind placebo and nocebo effects are the focus of much theoretical and empirical work. Several potential mechanisms have been suggested. Below, we discuss the three psychological pathways that are currently the most widely discussed as mechanisms of the placebo effect: response expectancies, mindsets, and conditioning. We discuss the way artifacts might harness placebo effects via each of these mechanisms after first introducing the mechanism in greater detail. It is important to note that it is the role of artifacts in eliciting these cognitive mechanisms that, we postulate, engenders health effects rather than the mere presence of artifacts *per se*. We address the link between artifacts and mindsets and expectancies together, since the latter two concepts are highly related. We emphasize that artifacts might exert effects via other placebogenic mechanisms, including embodiment (Ongaro and Kaptchuk, 2019), social observational learning (Colloca and Benedetti, 2009), the “somatic focus” model (Alfano, 2015), and the Bayesian model of perceptual decision (Geuter et al., 2017). Since these mechanisms have been subject to less sustained empirical research, they lie beyond the scope of this paper.

### Response Expectancies

Response expectancies can elicit beneficial effects as a result of a patient’s beliefs that a treatment or intervention will be effective (e.g., Kirsch, 1985, 2018). Some psychologists appear to define response expectancies as consciously held beliefs or expectations. However, recent research implies a broader interpretation of expectations as encompassing non-verbal, implicit, and non-conscious “beliefs” and dispositions (Geers et al., 2005; Wellman and Geers, 2009; Jensen et al., 2012, 2014).

There is a rich body of research on the purported role of response expectancy in placebo studies. One example is anticipating a reduction in pain after the application of a specific cream described as having analgesic properties. If an individual experiences analgesia from this cream, yet the substance is actually inert, it is proposed that response expectancy has mediated the placebo effect. Several experiments investigating the role of response expectancy on placebo effects have been conducted. Benedetti et al. (1999) induced pain in healthy subjects through a subcutaneous capsaicin injection in the participant’s right foot, left foot, right hand, and left hand. When an inert topical cream was applied to the left hand (one experimental condition) or right hand and left foot (another experimental condition), participants expected to experience less pain in the affected areas with no change in the unaffected areas. When pain ratings were taken, actual pain intensity was correlated with these expectations.

### Mindsets

More recently, advancing a new research agendum, health psychologists proposed a “new framework for harnessing placebo effects in medicine” (Zion and Crum, 2018). Bearing resemblance to earlier terms in social psychology – “gestalt” and “schema” – and to “paradigms” in social sciences and philosophy, the authors describe *mindsets* as “lenses or frames of mind that orient individuals to particular sets of associations and expectations” (p. 147) (Zion and Crum, 2018). Differentiating this mechanism from response expectancies, Zion and Crum state that, “Expectations are specific beliefs about future events. *Mindsets* are a more general psychological construal that orient an individual to a number of mindset-consistent expectations” (p. 147).

Arguing that the provider-patient interaction can influence mindsets, and thereby placebo effects (Zion and Crum, 2018), researchers working from this perspective have constructively focused on perceptions of clinicians’ competence and empathy (see Howe et al., 2019 for a review). This research builds on, and refines, an earlier study by Kaptchuk et al. (2008), which showed that placebo acupuncture for Irritable Bowel Syndrome delivered by an especially warm and empathetic provider resulted in more relief compared to placebo acupuncture delivered by a less empathetic – or “businesslike” – provider. More recently, Howe et al. (2017) examined the role of warmth and competence in influencing placebo effects. The authors induced an allergic reaction in volunteer participants, and applied a sham topical cream. The provider interaction differed on three dimensions: warmth (high vs. low), competence (high vs. low), and expectation (high vs. low). Of particular relevance to this paper, the authors changed artifacts in line with the “competence” manipulation. In the high competence condition, posters with warm images were displayed and the room was “organized, neat and clean” (p. 1076). In the low competence condition, posters were not shown, and the room was disorganized with “papers scattered on floor” (p. 1076). Results showed that participants with positive expectations of allergy relief, and who were treated by a practitioner high in warmth and competence, experienced a reduction in their allergic reaction, as measured by the wheal size. This study provides initial evidence for the notion that artifacts might influence placebo effects. However, in the competence manipulation, other cues that lie outside of artifacts were also varied (e.g., the presence or absence of eye contact, putting the blood pressure cuff on correctly or incorrectly). Thus, the impact of artifacts in this study was confounded with the effects of other cues, and further research is needed to systematically examine the potential contribution of artifacts to placebo effects.

### Artifacts and Expectancies and Mindsets

The artifacts discussed in this paper often signal certain attitudes about providers. These attitudes are likely tied to both mindsets and treatment-relevant expectancies. For instance, one might have the mindset: “Well-dressed people are good at their job.” After seeing a formally attired doctor, this could translate to the expectancy: “Dr. X will help me get better.” Similarly, observing professional credentials conspicuously on display might lead to higher expectancies. In terms of nocebo effects, the mindset, “A shabby or uninviting room means the physician is not up to the job” could be linked to the expectancies “My doctor will not know what is wrong with me (incompetence) and/or will not listen attentively (unempathetic)” (additional examples of the proposed relationship between mindsets and expectancies are provided by Zion and Crum, 2018). Teasing apart the role of specific artifacts in mediating perceptions of physician competence and/or empathy, we suggest, would be a valuable task for future empirical research in placebo studies.

### Classical Conditioning

Classically conditioned placebo effects occur when symptom reduction in response to a placebo has been learned through pairings. In some experimental designs, placebo conditioning consists of two phases. In the conditioning phase, one cream (the placebo) is presented as an effective analgesic. Afterward, a painful stimulation is provided on both the placebo-treated and the non-treated skin sites. Participants are then informed that the stimulus intensity (e.g., heat pain) will be the same on both sites, whereas in fact it is deliberately reduced for the placebo-treated site in order to reinforce the experience of pain relief. During the final test phase, equivalent levels of painful simulations are performed on both sites. Researchers employing this paradigm have observed analgesia in the placebo-treated region in a number of different studies (e.g., Voudouris et al., 1985; Montgomery and Kirsch, 1997; Amanzio and Benedetti, 1999; Benedetti et al., 2007). Although much of this research does not evaluate classical conditioning fully independently of response expectancies, since participants believe they are receiving an analgesic agent, other work (e.g., Jensen et al., 2015; Ba̧bel et al., 2017) has confirmed that conditioning alone can elicit placebo effects.

### Artifacts and Classical Conditioning

How might the influence of artifacts be mediated by classical conditioning? In the West, at least, white coats are synonymous with medical care. Conceivably, they may also play a role in conditioned health responses within clinical encounters. Learned associations between a stethoscope (i.e., conditioned stimulus) and (for example) positive health outcomes, might result in symptom improvement (i.e., a conditioned healthcare response) if the neutral, or conditioned, stimulus evokes an unconscious response as a result of the repeated pairing. The negative counterpart to this – nocebo effects – arise when repeated learned associations between a neutral stimulus and adverse responses occur. For example, in the past, one might have had negative conditioned associations, with physicians wearing white coats or scrubs, or with the distinctive appearance of a particular clinical environment. As a result of this conditioning, the white coat/scrubs/appearance of the current clinical environment, could itself evoke anxiety in the present. To illustrate: while its mechanisms have not been related to nocebo research, “white coat hypertension” might be a nocebo effect that results from conditioned health anxieties when doctors perform medical tests in recognizable professional apparel which, in many health settings, is typically a white coat. It should be emphasized that classical conditioning in these contexts is strictly a result of repeated pairings over-time, consistent with the animal model work from Pavlov (1927) and others (Rescorla, 1988). Some of the placebo conditioning studies mentioned above (e.g., Montgomery and Kirsch, 1997) employed a combination of verbal cues and repeated pairings, which might over-estimate the real-world impact of conditioning in this context. Nonetheless, since analgesic and hyperalgesic conditioning to pain stimuli can occur without conscious awareness (Jensen et al., 2012, 2015), verbal instructions do not seem necessary for conditioned placebo effects.

## Next Steps: Exploring the Role of Artifacts and Placebo Effects

To better explore the connections between artifacts and placebo effects, we suggest that future studies investigate whether manipulation of artifacts – such as cues of clothing and aesthetics – influences the size of placebo effects. Based on the conceptual outline summarized above, we suggest that a preliminary experimental framework explore a classical conditioning paradigm to manipulate whether conspicuous artifacts or the apparel of a physician augment or diminish placebo effects. While much of the research we cover focuses on subjective outcomes, research ought to consider incorporating objective measures (e.g., behavior) [e.g., Zech et al. (2019)]. In addition, as we describe in more detail below, we suggest there may be significant promise in focusing on how artifacts influence expectancies and mindsets in clinical encounters. For example, we hypothesize that for certain placebo-effect responsive conditions and symptoms, patients may be more likely to experience beneficial effects if they encounter formally attired, versus casually dressed physicians, and if they encounter higher quality versus lower quality office furnishings and waiting areas.

In order to examine whether the magnitude of placebo effects is influenced by artifacts, we advance three research designs (Table 1). First, future research might investigate whether physician behavior changes naturalistically in response to the manipulations. For instance, if a provider is exposed to enriched artifacts, he or she may non-consciously interact with patients differently, thereby enhancing placebo effects. Second, investigators could provide the same inert^1^ treatment to all participants (such as sham-acupuncture) and vary the extent to which the use of artifacts is optimized, e.g., positive vs. neutral vs. negative. Based on the hypothesis presented in this article, we predict the following pattern of magnitudes of placebo effects for the three groups: positive use of artifacts > neutral use of artifacts > negative use of artifacts. A final approach would utilize a variation of the Balanced Placebo Design with Enrichment (BPDE) proposed by Kube and Rief (2017). In this design, both the treatment being provided (such as drug vs. placebo) and the therapeutic setting (enriched vs. impoverished) is varied, thus allowing the examination of main and interaction effects. In particular, the treatment could be varied by comparing several conditions (e.g., drug vs. placebo, open-label placebo vs. deceptive placebo, or placebo vs. no treatment/usual care); with respect to artifacts, the therapeutic setting could be varied as described above (e.g., positive use of artifacts vs. neutral). This 2 × 2 design would enable researchers to examine whether a specific treatment “requires” a positive therapeutic setting to be effective. In these studies, at least two experimental conditions are required: one condition in which the use of artifacts is optimized, and one condition with neutral or even negative use of artifacts (e.g., physician being dressed casually or even “inappropriately”). More conditions could also be considered if the goal is to test the presence of a dose-dependent relationship. Formal mediation or moderation analyses could be used across these designs to examine the extent to which these artifacts are mediated or modulated by perceived warmth and competence (Howe et al., 2019). Researchers should also consider employing manipulation checks to verify that artifacts were indeed manipulated in the expected manner. This could be done with participants who were in the primary study, or with a pre-selected group of volunteers viewing images. While the former is preferable, it may not be relevant for unconscious effects.

**TABLE 1:** Artifacts and placebo effects: Future research directions.

**Study description**	**Study arms**	**Next step example**
Artifacts influence on provider behavior	(1) Enriched artifacts within medical setting (2) Neutral artifacts within medical setting (3) Impoverished artifacts within medical setting	Evaluate whether artifacts are associated with provider behavior change and increased placebo effects: e.g., do enriched artifacts cause doctors to engage in more eye contact or behave more empathically, as judged by blinded third-party raters
Artifact impact on placebo effect	(1) Sham treatment, enriched artifacts (2) Sham treatment, neutral artifacts (3) Sham treatment, impoverished artifacts	Evaluate whether sham acupuncture is more efficacious when delivered by a well-, versus neutrally-, versus poorly-dressed provider
Balanced placebo design with enrichment	(1) Verum treatment, enriched setting (2) Verum Treatment, Impoverished setting (3) Sham treatment, enriched setting (4) Sham treatment, Impoverished setting	Examine the interaction between treatment (verum versus sham) and office décor (well-furnished v. poorly furnished) on healthcare outcomes.

**Box 1. [b] Key suggestions and findings.**

**Prior research shows:**

• Artifacts in the clinical environment – such as clinician apparel, and office décor – can impact patient perceptions of healthcare providers, and also health outcomes.
• Placebo and nocebo effects are genuine psychobiological events that engage perceptual and cognitive processes to elicit, respectively, positive and negative health changes.
• Mechanisms of placebo and nocebo effects are thought to include response expectancies, mindsets, and classical conditioning.
• Research in placebo studies has focused on the nature of treatments, or the role of clinician communication, including information disclosures and socio-emotional cues, in eliciting placebo and nocebo effects.

**We suggest that:**

• Artifacts in clinical settings may modulate mechanisms of placebo and nocebo effects.
• Experimental research could be conducted to better understand how artifacts impact health outcomes, and be ethically harnessed in clinical contexts.

Further considerations that we have not elaborated on relate to mobile Health (or “mHealth”) including apps aimed at helping patients to manage their illnesses or symptoms. Artifactual features of mobile devices, for example, might augment the size of placebo effects (Torous and Firth, 2016; Pontén et al., 2019). Similarly, incidental features in the design of mHealth apps, for example, how sophisticated the imagery or graphic design are – might also be conceived of as non-medical physical artifacts – that might augment (or diminish) placebo effects. This is especially important as there are measurable effects of smartphone-based interventions for several health conditions, such as depression (Firth et al., 2017), and owing to their potential, the U.S. Food and Drug Administration (FDA) has already approved some health applications as digital-based drugs (Waltz, 2018). Hence, we suggest that design features of eHeath innovations may influence artifactual placebo effects (Torous and Firth, 2016), a consideration that warrants further exploration.

## Ethical Implications

If the theory proposed in this conceptual analysis is borne out by empirical research, patients accessing health centers with upscale décor and furnishings may conceivably experience a boost in their clinical outcomes across a range of conditions and symptoms for which the placebo effect is relevant. In such a scenario, artifactual differences across clinical environments might be responsible for unequal distributions of placebo effects. Dependent on empirical findings, whether any such differences constitute injustice in care, we suggest, will require further ethical analysis.

We acknowledge that it may seem curious to consider aesthetic or artifactual aspects of clinical environments as potentially important features of healthcare. However, the fact that such factors *are not* currently considered part of the toolkit that ought to be focused on during clinical education does not imply they *should not* be reclassified as such (Blease, 2012; Friesen and Blease, 2018). Indeed, if further empirical studies suggest that artifacts do play a role in placebo effects, this may prompt other ethical questions. For example, should patients be informed of the remedial effects of their aesthetic surroundings? In fee-for-service systems of healthcare, should clinicians be reimbursed for the therapeutic boost implemented by their beneficial aesthetic taste? Additional questions relate to determining the patient’s values in person-centered care. For example, if artifacts elicit different effects among different demographic or groups, this may incur challenging ethical and practical dilemmas about décor and attire, and whether aesthetic dimensions of clinical encounters can and should be matched to individual patients.

Finally, should aspects of care that make a real difference to patient outcomes be taught to clinicians? We flag up these issues to highlight that non-trivial practice implications that might arise, if research demonstrates that artifacts may modulate the size of placebo and nocebo effects.

## Limitations

The artifacts discussed in the present paper focused on décor and clothing. This is not to suggest research should be limited to these aspects of healthcare but rather that they might present an important entry point for considering the role of artifacts in placebo studies. Furthermore, although we have specifically concentrated on recognizable artifacts that might modulate mechanisms of placebo and nocebo effects other physical aspects should not be ignored. Ambient features such as lighting, temperature, scent, and sound can certainly contribute to the sum of environmental effects (Harris et al., 2002; Fenko and Loock, 2014), and may also influence placebo and nocebo effects. Additionally, there may be cultural variations in how artifacts elicit effects. Depending on an individual’s background and life experiences, diplomas and particular styles of interior, for example, may evoke different responses (also see Moerman, 2002 for a relevant review).

## Conclusion

Artifacts have been studied at some length in social psychology but have not yet been the subject of systematic investigation in the field of placebo studies. Building on findings in psychology, and current research in placebo studies, we suggest that it is fruitful to connect these fields of research (see Box 1). If empirical research supports the hypotheses forwarded in this paper, enhanced consideration of aesthetic features could lead to important benefits for patients. We suggest that even apparently trivial measures to maximize patient outcomes may be worthwhile. Optimization of artifacts in clinical settings represents one such promising possibility.

## Author Contributions

CB conceived the manuscript. MB, CB, CL, and TK wrote the first draft. MB, CB, and CL revised the first draft. MB, SB, SS-F, and CB performed the revisions after peer-review. All authors contributed to the article and approved the submitted version.

## Conflict of Interest

The authors declare that the research was conducted in the absence of any commercial or financial relationships that could be construed as a potential conflict of interest.

## Footnotes

1 One could also examine the impact of artifacts on verum treatment, but our paper is mostly concerned with the way artifacts influence placebo effects.
